# Case Report: Amyand's Hernia, a Rare Form of Inguinal Hernia

**DOI:** 10.7759/cureus.117002

**Published:** 2026-09-27

**Authors:** Gulvanich Pipatvanichgul

**Affiliations:** 1 Interventional Radiology, Rajavithi Hospital, Bangkok, THA

**Keywords:** amyand’s hernia, appendicitis, appendix, groin hernia, inguinal hernia

## Abstract

A herniation sac containing the appendix, with or without inflammation, is known as Amyand’s hernia, which is one of the rarest types of inguinal hernia. The condition is predominantly diagnosed during surgery, as preoperative identification via diagnostic imaging remains uncommon.

This report presents two cases of Amyand’s hernia. The first case was an 80-year-old male with clinical signs and symptoms of a right inguinal hernia. The ultrasound images confirm the diagnosis of Amyand’s hernia. Based on established classification systems, management involved hernia reduction and mesh repair without appendectomy. The second case was a 63-year-old male with a history of rectal cancer. A CT scan revealed an incidental finding of the appendix within the right inguinal canal without evidence of inflammation, leading to a diagnosis of Amyand’s hernia.

Preoperative diagnosis remains essential for surgical planning to improve surgical success rates and minimize postoperative complications. Therefore, documenting Amyand’s hernia in the radiology report plays a pivotal role in optimizing patient management.

## Introduction

An inguinal hernia is a condition that is caused by a weak abdominal wall, resulting in protrusion of intra-abdominal tissue or organ into the inguinal canal. Common presentations of this condition are a visible or palpable lump in the groin, groin pain or discomfort, or scrotal swelling.

Common structures in the herniation include omentum and small bowel. Unusual structures are the appendix, ovary, urinary bladder, and colon [1].

Herniation sac containing appendix with or without inflammation is known as Amyand’s hernia, which is one of the rarest types of inguinal hernia. The prevalence of Amyand’s hernia is about 0.28% to 0.37% of all inguinal hernias, and Amyand’s hernia with appendicitis is found in only about 0.1% of all appendicitis cases [2,3]

Most cases of Amyand’s hernia are diagnosed intraoperatively. Only about 17.2% of Amyand’s hernia cases get the diagnosis before operation by using CT scan or ultrasound [3]. Preoperative diagnosis is important for operative planning to increase the success rate of the surgery and decrease postoperative complications. Imaging can demonstrate the content of the hernial sac and the condition of the appendix within it, including whether it is inflamed, to help decide and plan for an appendectomy. Therefore, the meticulous documentation of an Amyand’s hernia within the radiologist's formal report is a critical step in patient management. In clinical scenarios where a patient has a documented radiological history of an appendix positioned near or within the inguinal canal, any subsequent presentation of inguinal hernia signs and symptoms warrants heightened clinical suspicion. Although these clinical manifestations remain inherently non-specific and cannot be definitively differentiated from conventional inguinal hernias through physical examination alone, recognizing the presence of the appendix alters the therapeutic paradigm. Consequently, the surgical and treatment planning for these individuals must be carefully tailored and may differ substantially from the standard management protocols applied to more common types of inguinal hernias.

## Case presentation

Case 1

An 80-year-old male with a relevant surgical history of a left inguinal hernia repair performed in 2022 presented to our hospital in early 2025. His chief complaint was a painless, palpable mass in the right groin region, which had been gradually noticeable over a one-month duration. Clinical history revealed that the mass became significantly more prominent during physical exertion, specifically upon heavy lifting, and was completely relieved when lying flat in a supine position.

Upon physical examination, a nontender, soft, and easily reducible mass was noted within the right inguinal area. There were no clinical signs suggestive of incarceration, strangulation, or bowel obstruction. Based on these classic clinical findings, a provisional diagnosis of a right inguinal hernia was established. To confirm the diagnosis and further evaluate the contents of the hernial sac, the patient was promptly referred to the radiology department for a comprehensive ultrasound evaluation.

Imaging

Ultrasound evaluation of the palpable lesion within the right groin demonstrated a hernial sac. The sac contained a viable bowel loop exhibiting normal active peristalsis, immediately adjacent to a blind-ended tubular structure. This blind-ended structure was highly characteristic of the vermiform appendix and measured within normal limits. There was no demonstrable sonographic evidence of acute appendicular inflammation, such as wall thickening, increased vascularity on color Doppler, or periappendiceal fluid collection. These sonographic features strongly supported the preoperative diagnosis of an uncomplicated Amyand’s hernia (Figure 1).

**Figure 1:**
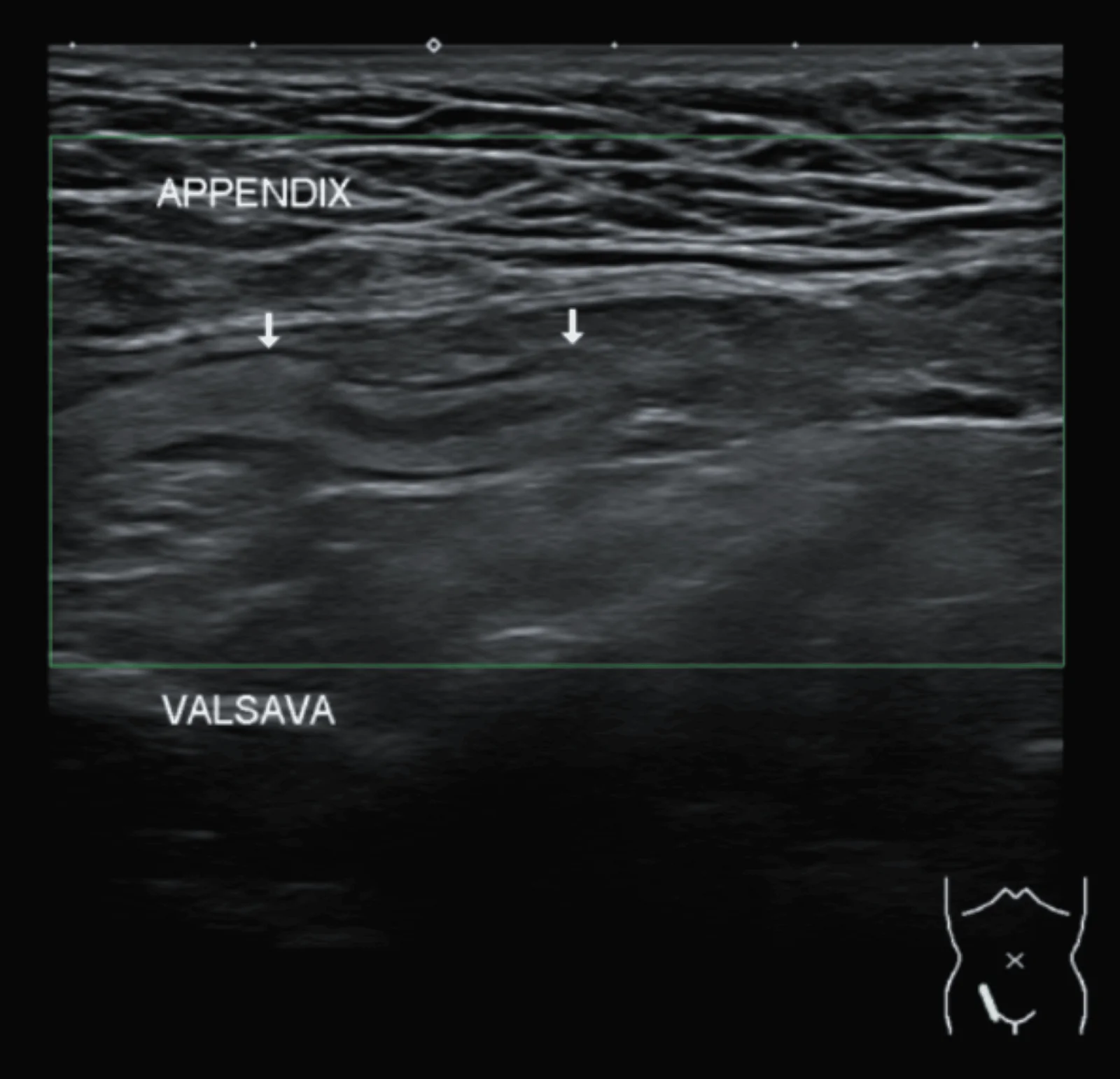
Blind end tubular structure in the right inguinal sac without evidence of inflammation.

Retrospective review of the patient's prior contrast-enhanced CT images of the abdomen from 2022 revealed a left inguinal hernia, which contained a segment of the sigmoid colon (Figure 2). Notably, at that time, the tip of the appendix was identified positioned immediately superior to the right deep inguinal ring, showing no extension into the inguinal canal or signs of herniation during that period (Figure 3).

**Figure 2:**
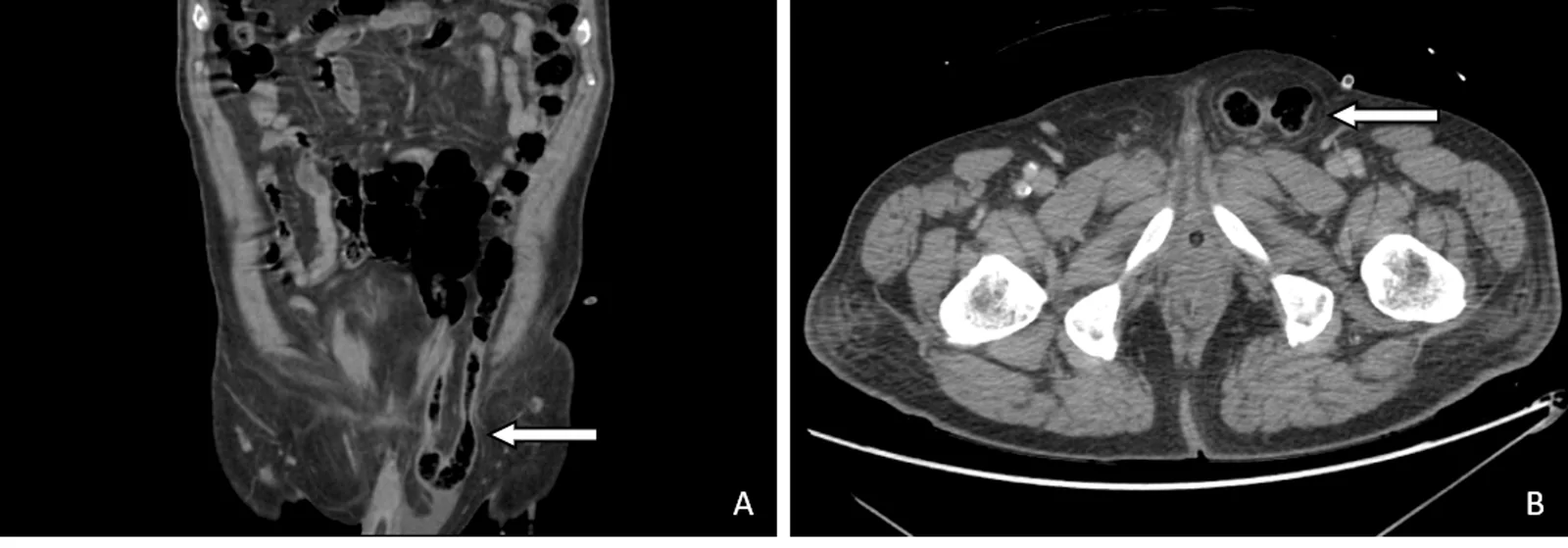
Coronal (A) and axial (B) CT images of the abdomen demonstrating the presence of a sigmoid colon loop inside the left inguinal hernia sac.

**Figure 3:**
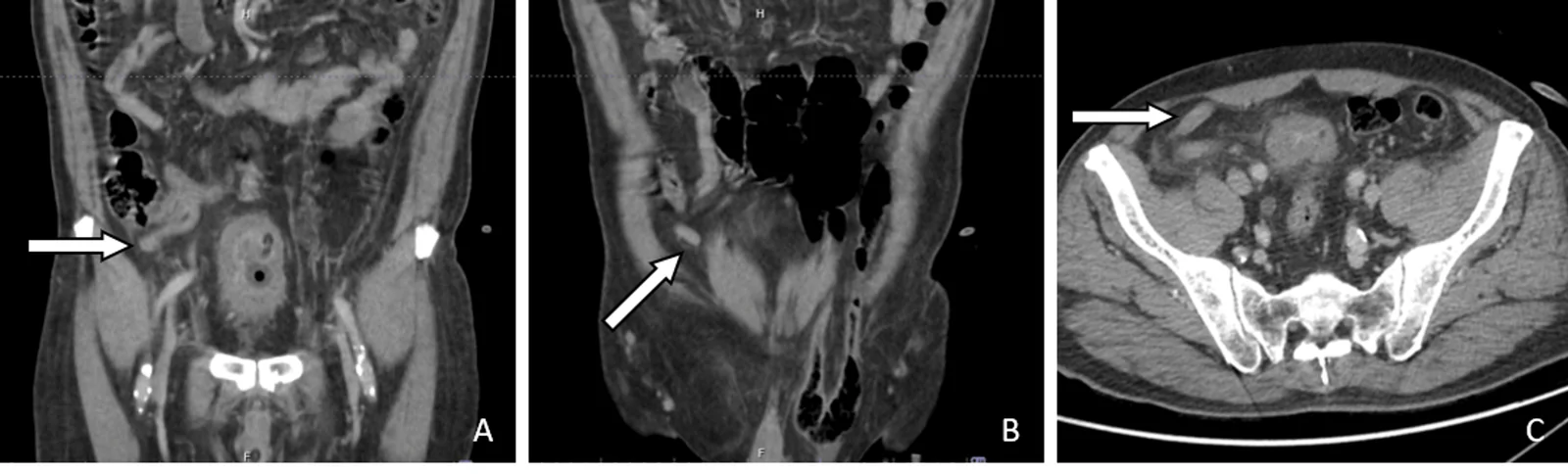
Coronal (A, B) and axial (C) CT images of the abdomen showing the appendix tip located just above the right deep inguinal ring.

Treatment

In May 2026, the patient successfully underwent a laparoscopic transabdominal preperitoneal inguinal hernia repair. Intraoperatively, the surgeon identified a non-inflamed appendix within the hernial sac. The surgical procedure was completed without any intraoperative or postoperative complications, and an appendectomy was deferred based on its condition.

Case 2

A 63-year-old male with a history of rectal cancer, who had previously undergone an open abdominoperineal resection in 2019, presented for a routine oncological follow-up in early 2026.

Laboratory investigations revealed a significant elevation in serum carcinoembryonic antigen levels. To investigate the potential disease progression, a comprehensive diagnostic workup, including a CT scan of the chest and abdomen, was promptly performed. 

The imaging studies demonstrated a local tumor recurrence, along with intra-abdominal lymph node and pulmonary metastases. Due to the advanced nature of the recurrent oncological disease, the patient was scheduled to initiate systemic chemotherapy.

Imaging

CT images of chest and abdomen confirmed the presence of a local tumor recurrence within the pelvic cavity. Additionally, the images revealed multiple enlarged intra-abdominal lymph nodes and bilateral pulmonary nodules, highly suspicious for distant metastases (Figure 4).

**Figure 4:**
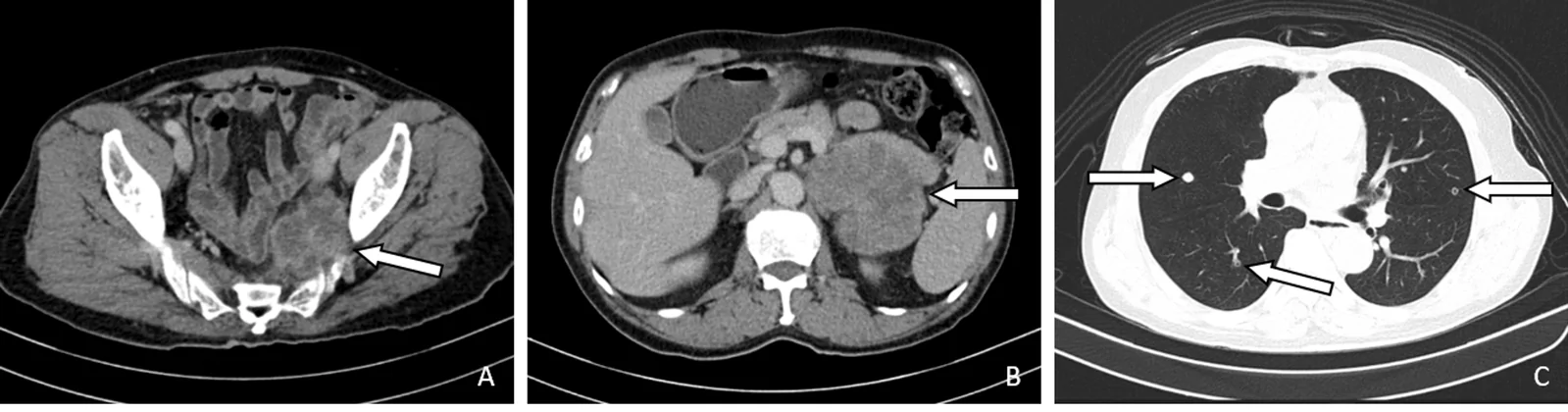
A lobulated mass in the left posterior pelvic sidewall (A), representing a potential local tumor recurrence. An enlarged intra-abdominal lymph node (B) and pulmonary nodules (C) are also shown.

Furthermore, CT imaging of the abdomen revealed an incidental finding of the appendix located within the right inguinal canal. The herniated appendix is measured up to 0.5 cm in caliber with no radiological evidence of acute inflammation, surrounding fat stranding, or fluid collection (Figure 5).

**Figure 5:**
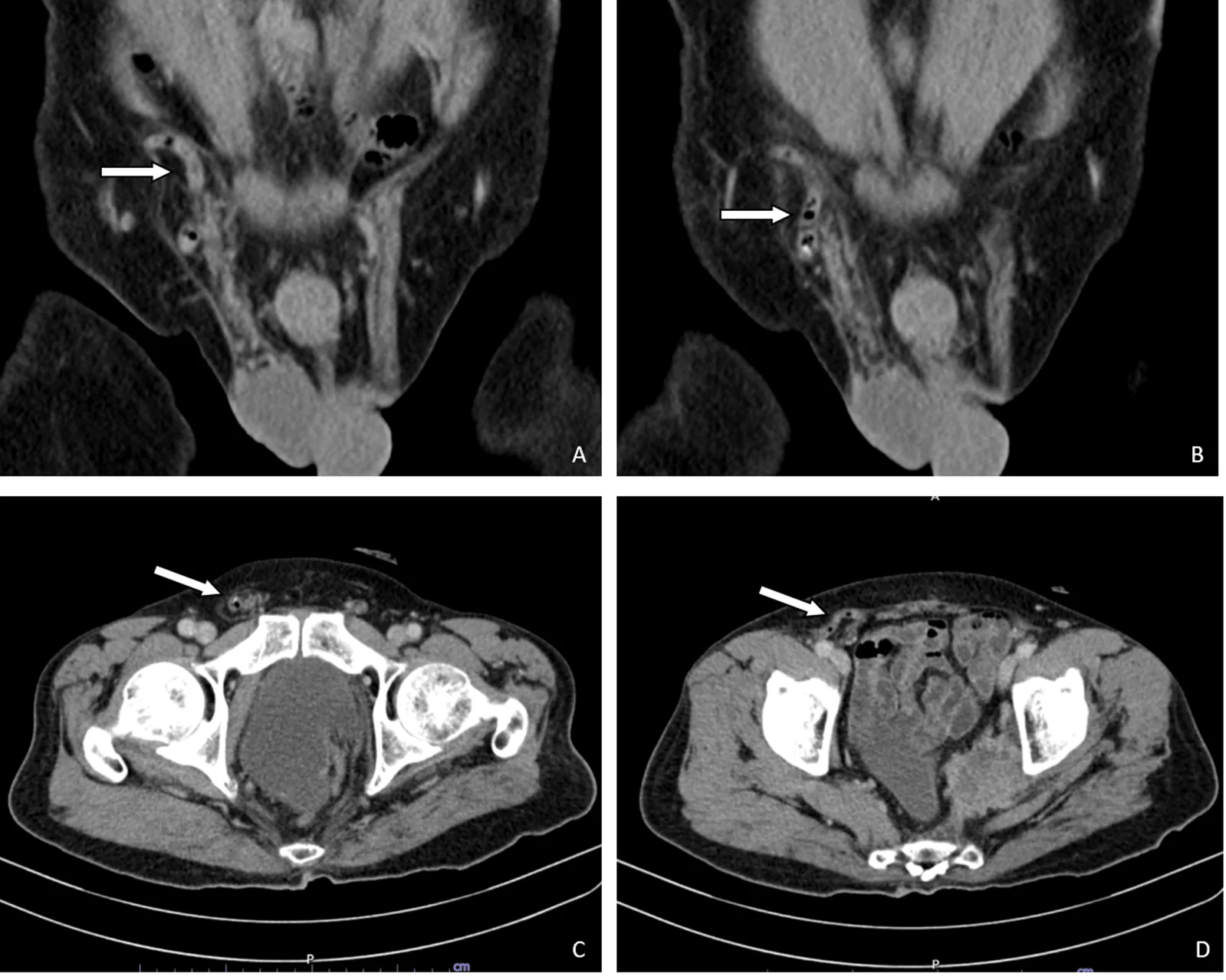
Coronal (A, B) and axial (C, D) CT images of the abdomen showing a non-inflamed appendix within the right inguinal canal.

Treatment

Regarding the management of the Amyand’s hernia, the patient remained completely asymptomatic with no clinical signs of hernia-related complications or incarceration. Given the advanced stage of his recurrent disease, the patient requested a formal referral to a medical center closer to his primary residence to undergo specialized palliative oncological treatment. Consequently, definitive plans for elective hernia repair were deferred at our hospital, prioritizing his immediate oncological care.

## Discussion

An inguinal hernia is a condition that is caused by a weak abdominal wall, resulting in protrusion of the intra-abdominal tissue or organs into the inguinal canal.

The presentation of an inguinal hernia is a palpable lump, pain, or discomfort in the groin or scrotal sac, which could be persistent or intermittent and correlated with increased intra-abdominal pressure. For the physical examination, the physician should observe or directly palpate bulging in the groin or scrotal sac with or without the Valsalva maneuver.

The diagnosis of inguinal hernia is usually made by the patient’s history, clinical presentation, and physical examination without imaging needed. However, some cases, especially in women, may require imaging to assist diagnosis and surgical planning.

The prevalence of Amyand’s hernia is about 0.28% to 0.37% of all inguinal hernias, and the prevalence is higher in pediatric patients, which may reach 1% [2]. A retrospective observational study with a literature review of Amyand’s hernia by Kaya et al. in 2024 [4] included inguinal hernias repaired in their clinic between January 2009 and March 2020. Amyand’s hernias were found in 13 of the 1,774 patients. All patients were male in the age range of 48 days to 2.5 years.

The clinical presentation of Amyand’s hernia resembles other types of inguinal hernia, making a specific clinical diagnosis challenging. Consequently, it is typically identified intraoperatively. Only about 17.2% of Amyand’s hernia cases are diagnosed before operation using CT scan or ultrasound [3].

Ultrasonography is the first-line imaging modality with sensitivity of 33% to 86% and specificity of 77% to 90% [5]. It can show real-time assessment of the movement of the herniation sac during examination. If ultrasound is negative or inconclusive and further evaluation is needed, then MRI or CT could be considered.

There are four subtypes of Amyand’s hernia regarding the clinical symptoms and the situation of the appendix [6]. Surgical planning is also different in each subtype, as shown in Table 1.

**Table 1:** Classification and management of Amyand’s hernia This classification guides surgical decision-making based on the condition of the appendix inside the inguinal hernia sac. Type 1 represents a healthy appendix where a synthetic mesh can be safely used. Types 2 and 3 involve acute appendicitis (without or with peritonitis, respectively), requiring primary tissue repair instead of mesh to avoid infection. Type 4 addresses cases with concomitant intra-abdominal or pelvic pathologies requiring secondary evaluation. The table was constructed by extracting and synthesizing the clinical criteria and management guidelines described in the study by Losanoff and Basson [6].

Classification	Description of the appendix within the hernia sac	Surgical management
Type 1	Uninflamed appendix	Hernia reduction with mesh hernioplasty (consider appendectomy in youth)
Type 2	Acute appendicitis without peritonitis	Appendectomy via the hernial defect, primary hernia repair without mesh
Type 3	Acute appendicitis with peritonitis	Exploratory laparotomy, appendectomy, and primary hernia repair without mesh
Type 4	Acute appendicitis with other concurrent pathology	Appendectomy, investigate and/or treat second pathology as appropriate

Amyand’s hernia with appendicitis is found in only about 0.1% of all appendicitis cases [2,3]. A case report of Amyand’s hernia complicated by appendicitis by Parekh et al. in 2021 [7] described an 81-year-old male who presented with an irreducible right inguinal mass. CT scan showed acute appendicitis within a right inguinal hernia. Another case report and literature review by Radboy et al. in 2023 [8] reported a 48-year-old male with a right groin protrusion and pain in the right lower quadrant of the abdomen and right groin. The ultrasound showed a 9-mm appendix in the right inguinal canal. These two patients underwent laparoscopic appendectomies and peritoneal defect repairs, with subsequent elective hernia repairs planned for a later date to avoid potential infectious complications associated with immediate mesh placement.

For the first case in this report, the patient exhibited classic clinical features of an inguinal hernia. Consequently, he was sent to the radiology department for an ultrasound to confirm the diagnosis. Notably, the sonographic evaluation unexpectedly demonstrated an uninflamed appendix situated inside the right inguinal hernial sac, thereby establishing a definitive preoperative diagnosis of an uncomplicated Amyand’s hernia.

According to the Losanoff and Basson classification of Amyand’s hernia, this case corresponds to type 1, characterized by the presence of a normal, uninflamed appendix within the hernia sac. Based on this established therapeutic guideline, the surgical management was tailored to perform a successful hernia reduction combined with a tension-free mesh repair, while an appendectomy was intentionally omitted to minimize unnecessary surgical trauma and avoid contaminating a clean surgical field.

A key educational insight from this case stems from the retrospective analysis of the patient's prior CT scan from 2022. The historical imaging revealed that the tip of the appendix was positioned immediately superior to the right deep inguinal ring. This anatomical configuration represented a close proximity of the appendix to the deep inguinal ring, capturing a potential precursor to an Amyand’s hernia before true herniation occurred. Therefore, this case highlights that when a patient presents with clinical signs of a right inguinal hernia, even in the absence of acute symptoms or initial imaging, clinicians and radiologists should maintain a high index of suspicion for an underlying Amyand’s hernia, particularly when historical imaging shows the appendix in close proximity to the hernial orifices.

In the second case of this report, the patient was entirely asymptomatic regarding the inguinal region, showcasing no clinical signs or symptoms of a hernia. The diagnostic evaluation, driven by an elevated serum tumor marker, was focused on investigating a suspected recurrence of rectal cancer. While the CT imaging confirmed the presence of a recurrent tumor with widespread metastasis, it simultaneously yielded an incidental finding of a normal, uninflamed appendix residing within the right inguinal canal. This unexpected radiological presentation established a concurrent diagnosis of a Type 1 Amyand’s hernia, according to the Losanoff and Basson classification.

 This case underscores a critical clinical scenario involving competing medical priorities. Although a Type 1 Amyand’s hernia typically warrants definitive surgical management via hernia reduction and mesh repair under elective conditions, the immediate therapeutic focus had to be shifted toward the patient's advanced oncological disease. Because the patient requested a formal transfer to another institution closer to his residence for specialized cancer therapy, no definitive management plan for the Amyand’s hernia was initiated at our hospital. This decision highlights the importance of clinical judgment in prioritizing life-threatening malignancies over asymptomatic, benign conditions, ensuring that immediate care is directed toward optimizing the patient's overall oncological outcome.

## Conclusions

The clinical presentation of Amyand’s hernia often mimics other types of inguinal hernias, making a specific clinical diagnosis challenging; consequently, it is typically identified intraoperatively. However, a preoperative diagnosis is crucial for surgical planning to optimize success rates and minimize postoperative complications.

This report highlights that in patients with a documented radiological history of an appendix positioned near or within the inguinal canal, any subsequent development of nonspecific inguinal symptoms should strongly prompt clinicians to consider a developing or incarcerated Amyand’s hernia. Once identified, clinical and surgical management should be strictly guided by the established Losanoff and Basson classification system to ensure tailored, evidence-based patient care. Ultimately, the meticulous documentation of Amyand’s hernia within radiology reports is of vital importance, even when encountered as an entirely incidental finding, as it serves as a critical communication tool that shapes definitive management strategies and improves overall patient outcomes.
